# Thermophysical Study of Pyridinium-Based Ionic Liquids Sharing Ions

**DOI:** 10.1021/acs.jced.1c00925

**Published:** 2022-02-10

**Authors:** Christian Reinado, Adrián Pelegrina, Miguel Sánchez-Rubio, Héctor Artigas, Carlos Lafuente

**Affiliations:** Departamento de Química Física, Facultad de Ciencias, Universidad de Zaragoza, 50009, Zaragoza, Spain

## Abstract

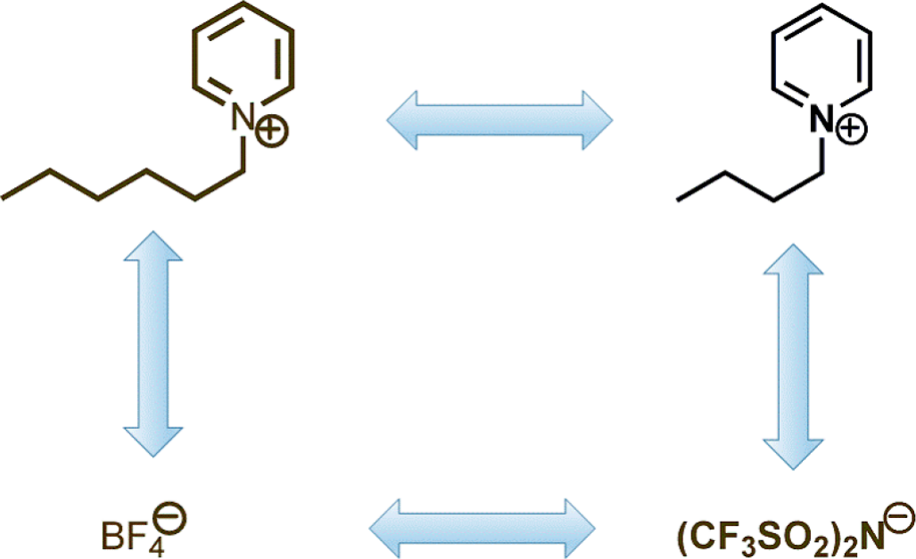

The thermophysical properties of three pyridinium-based ionic liquids sharing ions were measured at several temperatures (278.15–338.15) K and at atmospheric pressure (0.1 MPa). Three ionic liquids were studied: 1-butylpyridinium bis(trifluoromethyl-sulfonyl)imide, 1-hexylpyridinium bis(trifluoromethylsulfonyl)imide, and 1-hexylpyridinium tetrafluoroborate. The following thermophysical properties were measured: density, speed of sound, refractive index, surface tension, isobaric molar heat capacity, kinematic viscosity, and electrical conductivity. The thermophysical properties at atmospheric pressure were correlated with temperature, noting that the starting temperature for the speed of sound measurements depended on the ionic liquid. From these experimental results, some derived properties (isentropic compressibility, molar refraction, and dynamic viscosity) are calculated. These results together with those published previously for 1-butylpyridinium tetrafluoroborate are discussed.

## 1 Introduction

Ionic liquids are salts that are liquids at moderate temperatures (below 373 K). The physicochemical properties of ionic liquids can be tuned by combining different cations and anions. In fact, these physicochemical properties depend on the superposition of different molecular phenomena. Therefore, the determination of a comprehensive set of thermophysical properties for ionic liquids is useful to understand these molecular phenomena. Apart from the theoretical interest in these kinds of studies, the experimental data obtained are also very useful for applications in industry.

We report a set of thermophysical properties including thermodynamic, acoustic, optical, and transport properties of three pyridinium-based ionic liquids: 1-butylpyridinium bis(trifluoromethylsulfonyl)imide, [bpy][Tf_2_N], 1-hexylpyridinium bis(trifluoromethylsulfonyl)imide, [hpy][Tf_2_N], and 1-hexylpyridinium tetrafluoroborate, [hpy][BF_4_], over the temperature range (278.15–338.15) K with a temperature step of 2.5 K at atmospheric pressure (0.1 MPa), although the starting temperature for speed of sound measurements depends on ultrasound absorption of the ionic liquid.^1^ On the other hand, the melting temperature of [bpy][Tf_2_N] is 299 K,^2,3^ so its properties under this temperature correspond to the undercooled liquid. No metastability was observed in the undercooled liquid. The following properties were experimentally determined: density, speed of sound, refractive index, surface tension, isobaric molar heat capacity, kinematic viscosity, and electrical conductivity. Using these experimental data, we also determined the isentropic compressibility, molar refraction, and dynamic viscosity. Taking into account our previously published results for 1-butylpyridinium tetrafluoroborate,^4^ we can discuss the influence of both the alkyl chain length of the cation and the anion nature.

In a previous paper, the *ρpT* behavior of these ionic liquids was reported.^5^ The densities were measured using an Anton Paar DMA HP cell with an Anton Paar DMA 5000 acting as the evaluation unit. The experimentally obtained raw densities were corrected using estimated viscosities, according to the method proposed by Sanmamed et al.^6^ While the densities presented in this work were measured with an Anton Paar DSA 5000 that automatically corrects the viscosity errors in the density. The small differences between these two sets of density data could be due to both the uncertainty of the density measurements and the different viscosity corrections.

There are several papers^2,3,7−25^ in the literature reporting the thermophysical properties of these ionic liquids, especially for 1-butylpyridinium bis(trifluoromethyl-sulfonyl)imide. Density data appear in most of the works. There are also a considerable number of studies reporting dynamic viscosity and electrical conductivity data. Few data have been reported for the properties other than density for 1-hexylpyridinium bis(trifluoromethylsulfonyl)imide. For these two ionic liquids containing the bis(trifluoromethylsulfonyl)imide anion, the recent work of Dzida et al.^25^ can be highlighted, which only lacks dynamic viscosities. Finally, for 1-hexylpyridinium tetrafluoroborate, there are only three references reporting thermophysical properties (density and refractive index), in two of which the properties are presented at only one temperature.

## 2 Materials and Methods

The ionic liquids used in this study are presented in Table 1. To reduce the amount of water before use, the ionic liquids were dried under vacuum of 0.05 kPa for 24 h. The final water content was determined by a Karl Fisher titration employing an automatic titrator Crison KF 1S-2B. The combined expanded uncertainty in water content was ±10 ppm. After the experimental determination of the thermophysical properties, the water content was measured again. No significant increases in the water content were observed. On the other hand, the halides content was less than 100 ppm according to the supplier.

**Table 1:** Ionic Liquids

chemical name	CASRN	source	purity^a^^,^^b^ (mass fraction)	water content (ppm)
1-butylpyridinium bis((trifluoromethyl)sulfonyl)imide	187863-42-9	Iolitec	0.99	408
1-hexylpyridinium bis((trifluoromethyl)sulfonyl)imide	460983-97-5	Iolitec	0.99	104
1-hexylpyridinium tetrafluoroborate	474368-70-2	Iolitec	0.99	430

a As stated by the supplier by NMR analysis.

b Ionic liquids were dried under vacuum of 0.05 kPa for 24 h.

The density, ρ, and speed of sound, *u*, were determined simultaneously using an Anton Paar DSA 5000. The density was determined by the vibrating tube method and speed of sound by an acoustic, time-of-flight method,^1^ with a device frequency of approximately 3 MHz. The densimeter automatically corrects the viscosity errors in the density. The calibration was performed in the full temperature range (273.15–343.15) K following the instructions of Anton Paar with distilled deionized water and dry air. The combined expanded uncertainties in the density, and speed of sound, were ±10^–3^ g·cm^–3^ and ±0.5 m·s^–1^, respectively, and the standard uncertainty in temperature was 0.01 K. The refractive index was determined at a wavelength λ = 589.3 (sodium D line), *n*_D_, using standard Abbe refractometry with an Abbemat-HP Dr Kernchen refractometer. The refractometer was calibrated using distilled deionized water. The combined expanded uncertainty in the refractive index was ±5 × 10^–5^, and the standard uncertainty in temperature was 0.01 K. The surface tension, σ, was obtained using a drop volume tensiometer and a Lauda TVT-2 tensiometer. To maintain the temperature, a Lauda E-200 thermostat was used. The combined expanded uncertainty of the measurements was ±0.5 mN·m^–1^, and for temperature the standard uncertainty was 0.01 K. The isobaric molar heat capacity, *C*_p,m_, was measured by differential scanning calorimetry with a TA Instruments DSC Q2000 calorimeter using a heating rate of 20 K·min^–1^.^9^ A synthetic sapphire disk from TA Instruments (3.2 mm diameter × 0.4 mm thick) for hermetic pans was used as a reference standard, and the combined expanded uncertainty for the isobaric heat capacity determinations was ±10 J·mol^–1^·K^–1^ and the corresponding standard uncertainty for temperature was 0.005 K. The kinematic viscosity, ν, was determined using several Ubbelohde capillaries by means of a Schoot-Geräle AVS-440 automatic unit. The constants of the Ubbelohde capillaries were certified by Xylem Analytics Germany GmbH. The combined expanded uncertainty in the kinematic viscosities was ±5 mm·s^–1^. The electrical conductivity, κ, was obtained operating at alternating frequency (2 kHz) with a conductimeter Crison LPG31. Two aqueous solutions of KCl were used to calibrate the cell. The combined expanded uncertainty in the electrical conductivity was ±0.20 mS·cm^–1^. In both cases, the temperature was kept constant with a Schoot-Geräte CT thermostat (1150/2) with a standard uncertainty in temperature, 0.01 K.

## 3 Results

The thermophysical properties measured in this study along with some derived properties are reported in Table 2. The corresponding plots for these properties as a function of temperature are shown in Figures 1–4 and in the Supporting Information Figures S1–S5.

**Table 2:** Experimental Thermophysical Properties and some Derived Properties of the Ionic Liquids^a^

*T*/K	ρ/(g·cm^–3^)	*u*/(m·s^–1^)	κ_S_/TPa^–1^	*n*_D_	*R*_m_/(cm^3^·mol^–1^)	σ/(mN·m^–1^)	*C*_p.m_/(J·mol^–1^·K^–1^)	ν/(mm·s^–1^)	η/(mPa·s)	κ/(mS·cm^–1^)
1-Butylpyridinium Bis(trifluoromethyl-sulfonyl)imide										
278.15^b^	1.4676	1290.69	409.0			34.90	534	117.9	173.0	0.802
280.65^b^	1.4652	1285.05	413.3			34.75	537	101.4	148.6	1.002
283.15^b^	1.4629	1279.37	417.6	1.447251	76.081	34.65	540	87.2	127.6	1.202
285.65^b^	1.4605	1273.78	422.0	1.446510	76.096	34.55	543	75.8	110.7	1.413
288.15^b^	1.4581	1268.24	426.4	1.445748	76.107	34.40	544	66.1	96.3	1.663
290.65^b^	1.4558	1262.70	430.8	1.445004	76.118	34.25	547	58.2	84.7	1.953
293.15^b^	1.4536	1257.20	435.3	1.444254	76.120	34.15	550	51.0	74.1	2.24
295.65^b^	1.4513	1251.69	439.8	1.443501	76.132	34.00	553	46.7	67.7	2.63
298.15^b^	1.4487	1246.20	444.5	1.442759	76.157	33.85	556	41.4	60.0	2.98
300.65	1.4465	1240.77	449.1	1.442003	76.159	33.75	559	37.1	53.7	3.39
303.15	1.4440	1235.36	453.8	1.441262	76.180	33.65	562	32.6	47.1	3.77
305.65	1.4416	1229.97	458.5	1.440511	76.192	33.55	565	29.9	43.1	4.23
308.15	1.4393	1224.46	463.4	1.439762	76.201	33.45	567	27.0	38.8	4.70
310.65	1.4370	1219.24	468.1	1.439015	76.213	33.30	570	24.5	35.1	5.15
313.15	1.4347	1213.89	473.0	1.438264	76.224	33.15	573	22.8	32.7	5.74
315.65	1.4323	1208.55	478.0	1.437557	76.239	33.05	575	20.6	29.5	6.21
318.15	1.4300	1203.24	483.0	1.436780	76.247	32.95	578	19.2	27.4	6.85
320.65	1.4277	1197.91	488.1	1.436028	76.255	32.80	581	17.7	25.3	7.45
323.15	1.4254	1192.68	493.2	1.435270	76.262	32.65	583	16.3	23.2	8.00
325.65	1.4230	1187.37	498.4	1.434541	76.277	32.50	586	15.0	21.4	8.53
328.15	1.4207	1182.07	503.7	1.433795	76.288	32.40	589	13.9	19.8	9.27
330.65	1.4185	1176.81	509.1	1.433050	76.294	32.30	592	13.0	18.4	9.76
333.15	1.4161	1171.39	514.6	1.432333	76.309	32.15	595	12.3	17.5	10.50
335.65	1.4138	1166.30	520.0	1.431594	76.322	32.05	597	11.7	16.5	11.19
338.15	1.4112	1161.03	525.7	1.430865	76.348	31.90	600	11.0	15.5	11.91
1-Hexylpyridinium Bis(trifluoromethylsulfonyl)imide										
278.15	1.3996					32.60	603	189.9	265.7	0.442
280.65	1.3972					32.50	606	161.2	225.2	0.528
283.15	1.3950			1.449204	85.484	32.35	609	136.8	190.8	0.624
285.65	1.3927			1.448447	85.497	32.20	612	117.8	164.1	0.735
288.15	1.3905	1252.81	458.2	1.447685	85.509	32.05	615	102.1	142.0	0.860
290.65	1.3882	1247.31	463.0	1.446926	85.523	31.90	618	88.4	122.7	0.992
293.15	1.3860	1241.79	467.9	1.446162	85.535	31.75	621	76.9	106.6	1.132
295.65	1.3837	1236.26	472.9	1.445398	85.548	31.60	624	67.4	93.3	1.286
298.15	1.3815	1230.71	477.9	1.444649	85.562	31.45	626	59.6	82.3	1.464
300.65	1.3792	1225.14	483.1	1.443887	85.573	31.35	629	52.4	72.3	1.656
303.15	1.3769	1219.54	488.3	1.443125	85.586	31.20	632	47.1	64.8	1.867
305.65	1.3747	1213.96	493.6	1.442372	85.602	31.10	635	42.2	58.0	2.09
308.15	1.3724	1208.36	499.0	1.441596	85.613	30.95	638	37.8	51.9	2.33
310.65	1.3702	1202.78	504.5	1.440834	85.622	30.80	641	33.4	45.7	2.59
313.15	1.3679	1197.29	510.0	1.440065	85.634	30.70	644	31.1	42.6	2.94
315.65	1.3657	1191.78	515.5	1.439304	85.645	30.60	646	28.0	38.3	3.23
318.15	1.3635	1186.31	521.1	1.438528	85.652	30.45	649	25.6	34.9	3.55
320.65	1.3613	1180.75	526.9	1.437756	85.661	30.30	652	23.4	31.9	3.89
323.15	1.3590	1175.20	532.8	1.437017	85.677	30.15	655	21.4	29.1	4.23
325.65	1.3568	1169.65	538.7	1.436268	85.685	30.05	658	19.7	26.7	4.61
328.15	1.3546	1164.11	544.7	1.435506	85.694	29.95	661	18.1	24.6	5.00
330.65	1.3524	1158.58	550.9	1.434772	85.712	29.80	663	16.8	22.7	5.40
333.15	1.3502	1153.05	557.1	1.434033	85.724	29.65	666	15.5	21.0	5.81
335.65	1.3480	1147.45	563.4	1.433286	85.733	29.50	669	14.4	19.4	6.27
338.15	1.3458	1141.99	569.8	1.432555	85.746	29.35	672	13.4	18.1	6.72
1-Hexylpyridinium Tetrafluoroborate										
278.15	1.1679					41.30	495	1585	1851	0.115
280.65	1.1662					41.10	498	1252	1460	0.146
283.15	1.1645			1.452944	58.270	40.85	499	998.2	1162	0.186
285.65	1.1628			1.452273	58.281	40.75	502	802.1	932.7	0.233
288.15	1.1611			1.451596	58.291	40.50	503	651.0	755.9	0.288
290.65	1.1594			1.450922	58.301	40.40	505	531.7	616.4	0.355
293.15	1.1577			1.450233	58.309	40.20	507	438.9	508.1	0.431
295.65	1.1560			1.449559	58.320	40.05	509	363.5	420.2	0.521
298.15	1.1543			1.448888	58.331	39.85	511	306.8	354.1	0.624
300.65	1.1525			1.448206	58.340	39.65	513	264.7	305.1	0.742
303.15	1.1509	1527.61	372.4	1.447526	58.348	39.40	516	225.5	259.5	0.875
305.65	1.1491	1521.34	376.0	1.446848	58.359	39.25	518	192.6	221.3	1.017
308.15	1.1475	1515.08	379.6	1.446173	58.365	39.05	519	165.3	189.7	1.175
310.65	1.1459	1508.92	383.3	1.445491	58.369	38.90	521	143.0	163.9	1.358
313.15	1.1443	1502.80	387.0	1.444826	58.377	38.70	523	124.2	142.1	1.566
315.65	1.1427	1496.71	390.7	1.444153	58.383	38.60	526	108.6	124.1	1.798
318.15	1.1410	1490.66	394.4	1.443473	58.390	38.35	527	94.7	108.0	2.050
320.65	1.1394	1484.61	398.2	1.442804	58.397	38.20	529	84.5	96.3	2.330
323.15	1.1377	1478.58	402.0	1.442207	58.413	38.05	531	73.4	83.5	2.65
325.65	1.1361	1472.92	405.7	1.441530	58.419	37.90	533	65.7	74.6	3.02
328.15	1.1345	1466.81	409.7	1.440852	58.425	37.70	535	58.6	66.5	3.40
330.65	1.1329	1460.64	413.8	1.440175	58.431	37.50	537	53.1	60.1	3.80
333.15	1.1313	1454.29	418.1	1.439497	58.435	37.35	539	47.2	53.4	4.21
335.65	1.1296	1448.10	422.2	1.438820	58.441	37.15	542	42.7	48.2	4.65
338.15	1.1280	1441.90	426.4	1.438142	58.448	36.90	543	38.7	43.6	5.12

a Taken as a function of the temperature, *T*, at atmospheric pressure, *p* = 0.1 MPa: density, ρ, speed of sound, *u*, isentropic compressibility, κ_S_, refractive index, *n*_D_, molar refraction, *R*_m_, surface tension, σ, isobaric molar heat capacity, *C*_p,m_, kinematic viscosity, ν, dynamic viscosity, η, and electrical conductivity, κ. Standard uncertainties *u* are *u*(*T*) = 0.01 K, *u*(*p*) = 1 kPa, and the combined expanded uncertainties *U*_c_ are *U*_c_(ρ) = ±10^–3^ g·cm^–3^, *U*_c_(*u*) = ±0.5 m·s^–1^, *U*_c_(*n*_D_) = ±5 × 10^–5^, *U*_c_(σ) = ±0.5 mN·m^–1^, *U*_c_(C_p,m_) = ±10 J·mol^–1^·K^–1^, *U*_c_(ν) = ±5 mm·s^–1^, *U*_c_(κ) = ±0.20 mS·cm^–1^, with 0.95 level of confidence (*k* = 2).

b Undercooled liquid.

**Figure 1:**
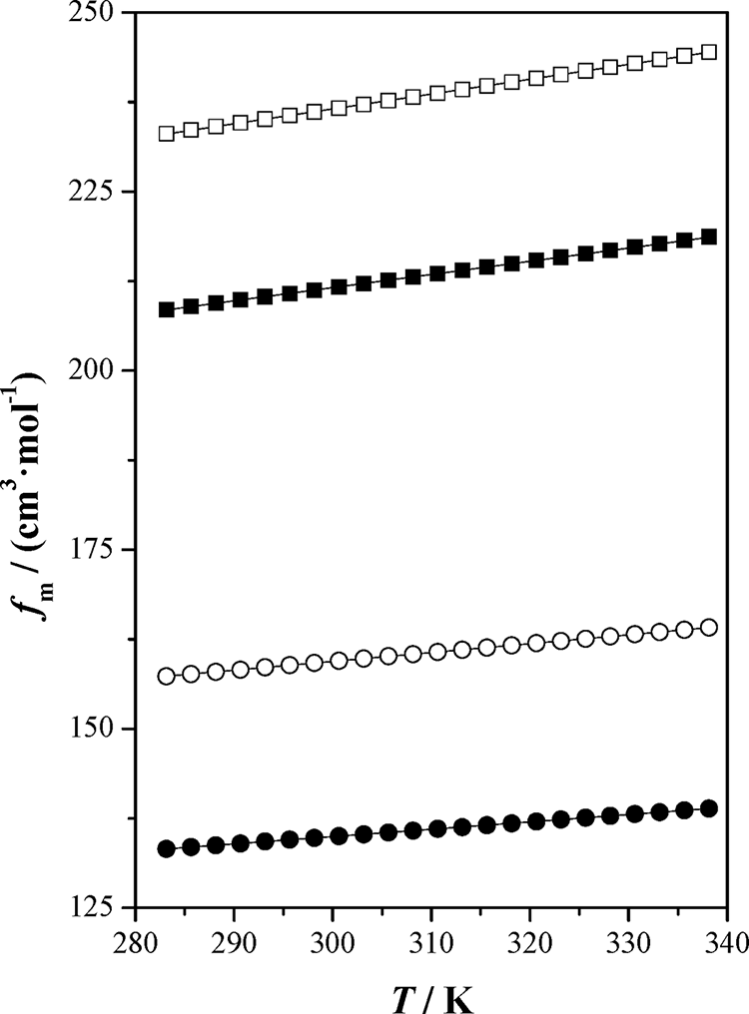
Free molar volume, *f*_m_, as a function of temperature, *T*, at *pp* = 0.1 MPa for the studied ionic liquids and [bpy][BF_4_]: (■) [bpy][Tf_2_N]; (●) [bpy][BF_4_] ref (4); (□) [hpy][Tf_2_N]; (○) [hpy][BF_4_]; (—) correlated values.

**Figure 2:**
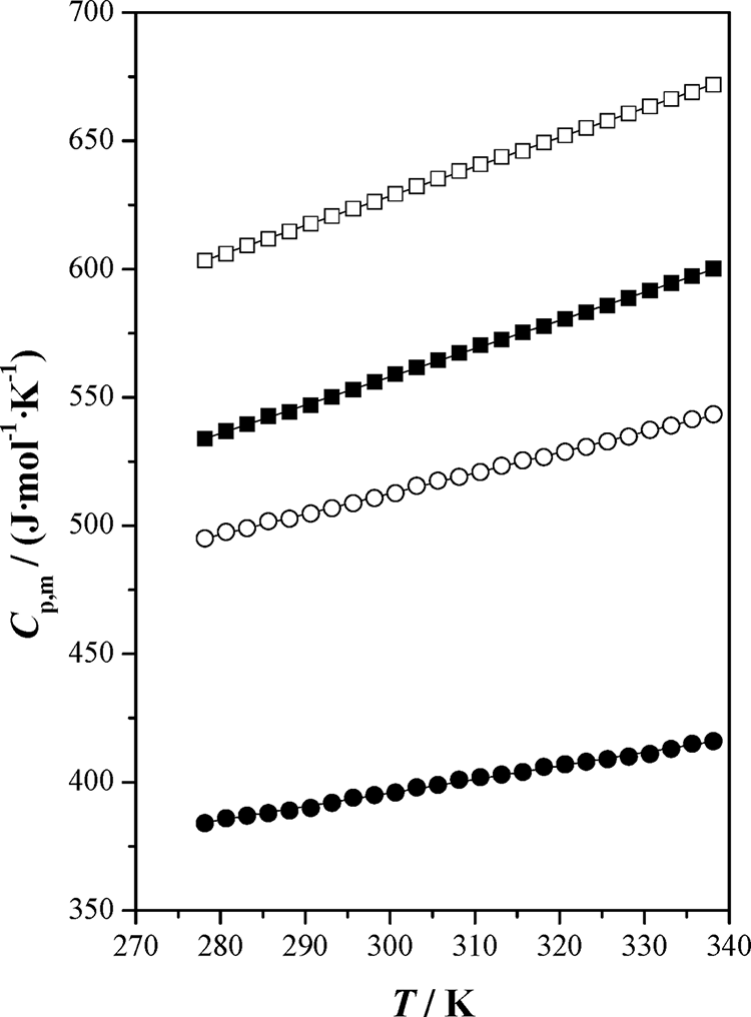
Isobaric molar heat capacity, *C*_p,m_, as a function of temperature, *T*, at *p* = 0.1 MPa for the studied ionic liquids and [bpy][BF_4_]: (■) [bpy][Tf_2_N]; (●) [bpy][BF_4_] ref (4); (□) [hpy][Tf_2_N]; (○) [hpy][BF_4_]; (—) correlated values.

**Figure 3:**
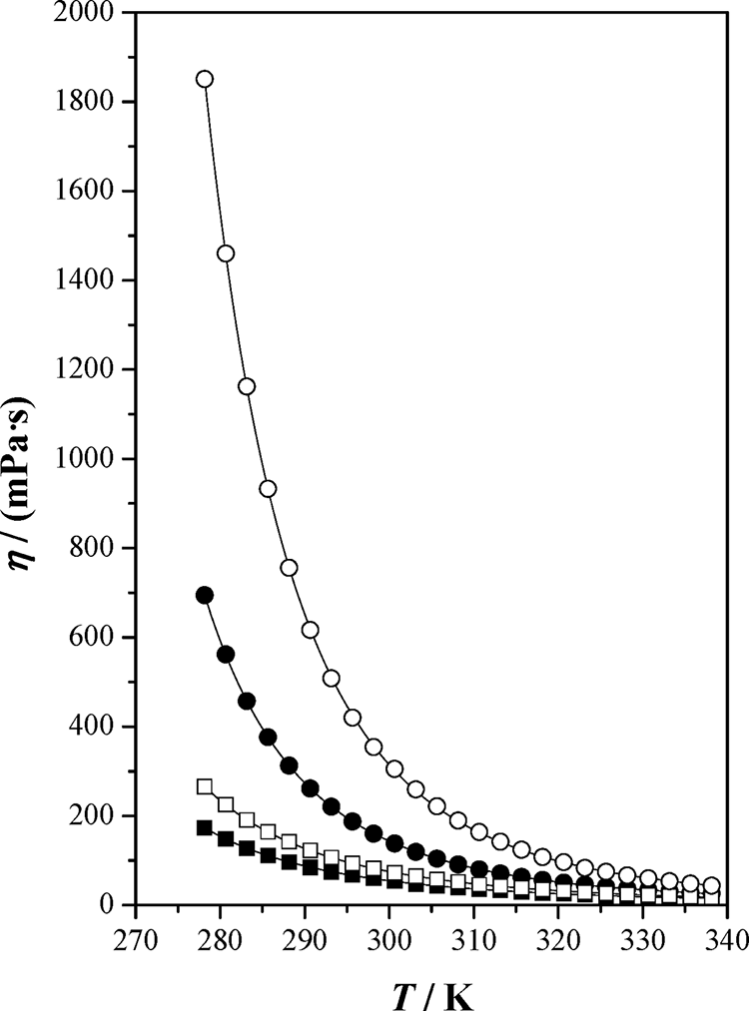
Dynamic viscosity, η, as a function of temperature, *T*, at *p* = 0.1 MPa for the studied ionic liquids and [bpy][BF_4_]: (■) [bpy][Tf_2_N]; (●) [bpy][BF_4_] ref (4); (□) [hpy][Tf_2_N]; (○) [hpy][BF_4_]; (—) correlated values.

**Figure 4:**
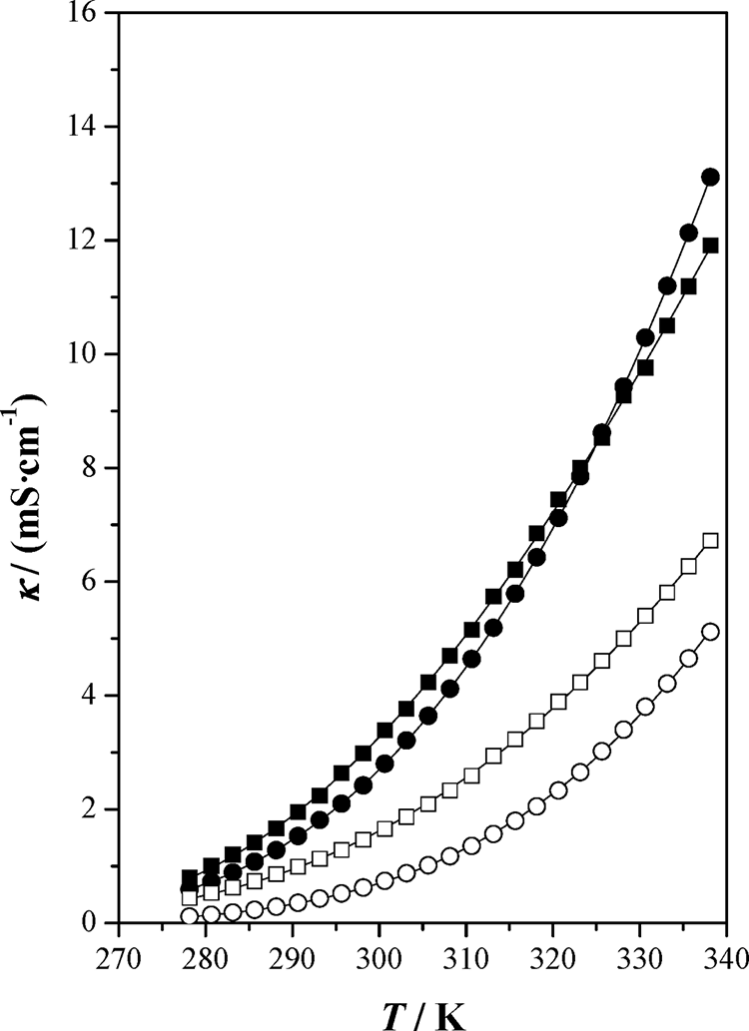
Electrical conductivity, κ, as a function of temperature, *T*, at *p* = 0.1 MPa for the studied ionic liquids and [bpy][BF_4_]: (■) [bpy][Tf_2_N]; (●) [bpy][BF_4_] ref (4); (□) [hpy][Tf_2_N]; (○) [hpy][BF_4_]; (—) correlated values.

The isentropic compressibility can be calculated from experimental ρ and *u* values, by means of the Newton–Laplace equation, *κ*_*S*_ = 1/(*ρu*^2^).

The molar refraction can be estimated through the Lorentz–Lorenz relation from the density (molar volume *V*_m_) and refractive index data, *R*_*m*_ = (*n*_*D*_^2^ – 1/*n*_*D*_^2^ + 2)*V*_*m*_.

The dynamic viscosity, η, can be obtained from kinematic viscosity, ν, and density, ρ, by means of η = ν·ρ.

For most of the properties, a linear dependence on the temperature can be established:
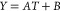
1where *Y* is the corresponding property and *A* and *B* are the fit parameters.

On the other hand, for the transport properties, dynamic viscosity and electrical conductivity, a different temperature behavior is observed, which can be described with the Vogel–Fulcher–Tammann equation:^26−28^
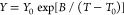
2where *Y* is η or κ, and *Y*_0_, *B*, and *T*_0_ are adjustable parameters.

To evaluate the goodness of fit, we used the absolute average relative deviation, AARD, defined as
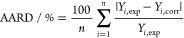
3The fitting parameters along with the corresponding absolute average relative deviations are given in Table 3.

**Table 3:** Fitting Parameters along with the Absolute Average Relative Deviations, AARD, for the Measured Properties

property	*A*	*B*	*C*	AARD/%
[bpy][Tf_2_N]				
ρ/(g·cm^–3^)	–0.000937	1.7282		0.01
*u*/(m·s^–1^)	–2.157	1889.65		0.03
*n*_D_	–0.000298	1.531725		0.01
σ/(mN·m^–1^)	–0.0495	48.65		0.05
*C*_p,m_/(J·mol·K^–1^)	1.100	228		0.05
η^a^/(mPa·s)	0.225	692	174.1	0.90
κ^b^/(mS·cm^–1^)	221.9	365.8	213.25	0.72
[hpy][Tf_2_N]				
ρ/(g·cm^–3^)	–0.000897	1.6488		0.01
*u*/(m·s^–1^)	–2.218	1891.96		0.00
*n*_D_	–0.000304	1.535187		0.01
σ/(mN·m^–1^)	–0.0537	47.50		0.09
*C*_p,m_/(J·mol·K^–1^)	1.144	285		0.04
η/(mPa·s)	0.115	870	165.9	0.47
κ/(mS·cm^–1^)	636.0	725.7	178.50	0.76
[hpy][BF_4_]				
ρ/(g·cm^–3^)	–0.000665	1.3525		0.01
*u*/(m·s^–1^)	–2.434	2265.33		0.02
*n*_D_	–0.000269	1.529019		0.01
σ/(mN·m^–1^)	–0.0718	61.25		0.09
*C*_p,m_/(J·mol·K^–1^)	0.801	272		0.06
η/(mPa·s)	0.069	1058	174.4	0.74
κ/(mS·cm^–1^)	3172.3	1033.7	177.20	0.92

a *A = η*_0_*; C = T*_0_.

b *A = κ*_0_*; C = T*_0_.

## 4 Discussion

The densities decrease when the temperature increases, and their values decrease following the sequence: [bpy][Tf2N] > [hpy][Tf2N] > [bpy][BF4] > [hpy][BF4]. The densities of the ILs containing the bis(trifluoromethylsulfonyl)imide anion are larger due to its greater weight with respect to the tetrafluoroborate anion.^29,30^ On the other hand, the presence of a shorter alkyl chain on the cation favors a better packing of ions, leading to higher densities. From the thermal behavior of the density (molar volume *V*_m_), the isobaric expansibility, α_p_, can be calculated, . The isobaric expansibility decreases with temperature. At *T* = 303.15 K the α_p_ values follow the sequence: [hpy][Tf2N] (6.556 kK^–1^) > [bpy][Tf2N] (6.531 kK^–1^) > [hpy][BF4] (5.808 kK^–1^) > [bpy][BF4] (5.687 kK^–1^). The α_p_ values increase with both the alkyl chain on the cation and the formula weight of the anion, a worsening packing of ions leads to higher free volume that favors the expansibility of the liquid.

The speed of sound decreases with temperature, following the *u* values with the order of [bpy][BF4] > [hpy][BF4] > [bpy][Tf2N] > [hpy][Tf2N]. The speed of sound increases with an efficient packing of ions. In this sense, both the presence of a shorter aliphatic chain and the lower size and asymmetry of the tetrafluoroborate anion compared with the bis(trifluoromethylsulfonyl)imide anion leads to the highest speed of sound for [bpy][BF4].

The isentropic compressibility property increases when the temperature increases, and the κ_S_ values show an opposite behavior to the speed of sound, with the isentropic compressibility of [bpy][BF4] being the lowest. This property is related to the volume variation against pressure changes, so if the ionic liquid is efficiently packed, its isentropic compressibility will be small.

The refractive indices decrease with temperature, and their values follow the sequence [hpy][BF4] > [bpy][BF4] > [hpy][Tf2N] > [bpy][Tf2N]. The ILs containing the tetrafluoroborate anion show higher *n*_D_ values, and for the same anion, the longer is the alkyl chain of the cation, the higher is the refractive index.

The molar refraction is related to the hard core molar volume.^31^ This property hardly changes with temperature in the considered temperature range, and *R*_m_ values decrease in the order of [hpy][Tf2N] > [bpy][Tf2N] > [hpy][BF4] > [bpy][BF4]. On the other hand, the free molar volume, *f*_m_, can be obtained by subtracting the molar refraction from the molar volume; in this case, the *f*_m_ values follow the sequence [hpy][Tf2N] > [bpy][Tf2N] > [hpy][BF4] > [bpy][BF4]. This sequence is the same as that presented by isentropic compressibility, so the poorly packed ILs present higher free molar volumes.

The surface tensions decrease with temperature, and the σ values follow the sequence [bpy][BF4] > [hpy][BF4] > [bpy][Tf2N] > [hpy][Tf2N]. Surface tension strongly depends on cohesive forces, and these cohesive interactions are stronger between pyridinium cations and the tetrafluoroborate anion than with the bis(trifluoromethylsulfonyl)imide anion. Among the cations, the cohesive interactions are weakest as the alkyl chain length increases due to the surface enrichment in the alkyl chains.^32,33^

The entropy of the surface per unit surface area, Δ*S*_*σ*_ = −(*∂σ*/*∂T*)_*p*_, and the enthalpy of the surface per unit surface area, Δ*H*_*σ*_ = σ – *T*(*∂σ*/*∂T*)_*p*_, are related to the surface tension behavior. The Δ*S*_σ_ values (ranging from 0.0718 mN·m^–1^·K^–1^ for [hpy][BF4] to 0.0495 mN·m^–1^·K^–1^ for [hpy][Tf2N]), these values are somewhat higher for the ILs containing [BF4]^−^ than for those containing [Tf2N]^−^. On the other hand, the alkyl chain length of the cation also influences the Δ*S*_σ_ values, which are slightly increased when the chain length is increased. With respect to Δ*H*_σ_ values that reflect the energy of the cohesive interactions, as can be expected, they follow the same sequence as for the surface tension values: at *T* = 303.15 K, [bpy][BF4] (65.20 mN·m^–1^) > [hpy][BF4] (61.20 mN·m^–1^) > [bpy][Tf2N] (48.65 mN·m^–1^) > [hpy][Tf2N] (47.45 mN·m^–1^).

The isobaric molar heat capacities increase with temperature, and their values decrease following the sequence [hpy][Tf2N] > [bpy][Tf2N] > [hpy][BF4] > [bpy][BF4]. It is known that when the number of atoms rises, the *C*_p,m_ values are higher due to the increased number of energy storage modes. However, this increase in *C*_p,m_ with the number of atoms is higher for ILs containing the tetrafluoroborate anion.

The dynamic viscosities decrease exponentially with temperature, and the η values decrease as follows [hpy][BF4] > [bpy][BF4] > [hpy][Tf2N] > [bpy][Tf2N]. In light of these results, the nature of the anion influences the viscosity behavior more than the length of the alkyl chain of the cation. The viscosity of [bpy][BF4] is greater than that of [hpy][Tf2N], probably due to the strong interaction between the tetrafluoroborate anion and the corresponding cation. Nevertheless, for each pair of ionic liquids, the length of the alkyl chain of the cation obviously increases the dynamic viscosity.

From the variation of the dynamic viscosity with temperature, the activation energy to viscosity process, *E*_*a*,η_, can be estimated using the expression,^34^*E*_*a*,η_ = *R*(∂ ln η/∂(1/*T*))_*p*_ where *R* is the ideal gas constant. These energy barriers follow the sequence [hpy][BF4] (51.4 kJ·mol^–1^) > [bpy][BF4] (45.5 kJ·mol^–1^) > [hpy][Tf2N] (37.0 kJ·mol^–1^) > [bpy][Tf2N] (33.6 kJ·mol^–1^), that is, the same sequence as that for the dynamic viscosities.

The electrical conductivities increase exponentially with temperature and decrease following the sequence [bpy][Tf_2_N] > [bpy][BF_4_] > [hpy][Tf_2_N] > [hpy][BF_4_], although from *T* ≈ 324 K, [bpy][BF_4_] shows the largest electrical conductivity. For this property, the alkyl chain length on the cation plays a major role, and the ILs containing the 1-butylpyridinium cation show the highest κ values. The influence of the anionic nature is not clear; at lower temperatures, ILs containing [Tf2N]^−^ present higher conductivity, but at elevated temperatures there is a change between [bpy][Tf_2_N] and [bpy][BF_4_]. In addition, the activation energy to the conductivity process was determined to follow the sequence [hpy][BF4] (52.3 kJ·mol^–1^) > [bpy][BF4] (43.2 kJ·mol^–1^) > [bpy][Tf2N] (38.3 kJ·mol^–1^), > [hpy][Tf2N] (37.6 kJ·mol^–1^). These values are very similar to the calculated values for the activation energy to the viscosity process.

Table 4 shows the comparison of our experimental results with literature values, expressed as AARD. It should be noted that the values of the thermophysical properties are influenced by the synthesis process and by the water content. This last factor is especially important in transport properties, dynamic viscosity, and electrical conductivity. In general, the agreement of our density results with those presented in the literature is reasonable, especially for [bpy][Tf2N] and [hpy][BF4] (AARD values lower than 0.1%), although for [bpy][Tf2N], two papers present higher deviations. For the case of [hpy][Tf2N], the deviations in density are larger than that for the other two ionic liquids, with the results from Bounsiar et al.^5^ and Bittner et al.^17^ being the closest to our results. In the work of Oliveira et al.,^10^ two sets of density values measured using a vibrating tube densimeter, are presented, one for dry liquids and the other one for liquids saturated with water, our experimental values fall between these two sets of values. On the other hand, with regard to the work of Liu et al.,^14^ it can be noted that the densities were measured by a Westphal balance instead of the usual vibrating tube densimeter. For the rest of the properties of the ILs studied, for which we have indicated that there are fewer studies, the concordance is also reasonable; for the surface tension, the biggest deviation of 7.6% occurs with the data of Bittner et al.,^17^ and for the dynamic viscosity, the largest deviation of 16% is shown by the work of Oliveira et al.^10^ It can be outlined that the largest deviations occur in the case of electrical conductivities with an average deviation of 12%, and these deviations are higher at low temperatures where the conductivities are smaller. For these low electrical conductivities, small conductivity differences lead to high AARD values. With respect to the recent study of Dzida et al.^25^ the concordance of both sets of thermophysical properties is satisfactory except for the electrical conductivity with deviations of approximately 24%, our electrical conductivities are higher probably due to the higher water content of our liquid. In the Supporting Information the figures showing the relative deviations, RD, RD = 100(*Y*_*i*,exp_ – *Y*_*i*,lit_)/*Y*_*i*,exp_ are presented. For most properties, literature values can be found with positive and negative deviations from our experimental values. It can be outlined that for [hpy][Tf2N] all the literature densities are somewhat greater than ours.

**Table 4:** Absolute Average Relative Deviations, AARD (%), between our Experimental and Literature Data

ref	*T*/K	ρ/(g·cm^–3^)	*u*/(m·s^–1^)	*n*_D_	σ/(mN·m^–1^)	*C*_p.m_/(J·mol·K^–1^)	η/(mPa·s)	κ/(mS·cm^–1^)
1-Butylpyridinium Bis(trifluoromethylsulfonyl)imide								
Tokuda et al.^2^	283–333	0.02					2.4	5.6
Liu et al.^3^	298.15–338.15	0.51			1.4			
Bounsiar et al.^5^	303.15–338.15	0.03						
Noda et al.^7^	293–313	0.03					6.0	
Diedrichs et al.^9^	330.15–338.15					1.2		
Oliveira et al.^10^	278.15–338.15	0.01					2.4	
Yunus et al.^11^	293.15–338.15	0.07		0.07			0.9	
Liu et al.^13^	283.15–338.15						3.7	6.3
Zhang et al.^15^	283.15–313.15							7.3
Bittner et al.^17^	293.15–323.15	0.37			3.1		1.4	
Larriba et al.^18^	303.15–333.15	0.01		0.02			3.4	
Zeng et al.^21^	293.15–338.15	0.02					1.3	
Santos et al.^22^	288.15–338.15	0.03						
Nazet et al.^23^	278.15–338.15	0.01		0.01			2.8	9.3
Yebra et al.^24^	283.15–333.15	0.02	0.4	0.04				
Dzida et al.^25^	283.15–338.15	0.01	0.1	0.01	3.6	3.7		24
1-Hexylpyridinium Bis(trifluoromethylsulfonyl)imide								
Bounsiar et al.^5^	283.15–338.15	0.04						
Oliveira et al.^10^	278.15–338.15	0.93					16	
Zhang et al.^15^	283.15–313.15							12
Bittner et al.^17^	293.15–323.15	0.11			7.6			
Dzida et al.^25^	283.15–338.15	0.12	0.9	0.08	0.9	4.9		19
Crosthwaite et al.^8^	283–323					2.9	1.1	
Liu et al.^14^	283.15–338.15	0.46			1.5		1.2	11
Bahadur et al.^20^	293.15–333.15	0.24						
1-Hexylpyridinium tetrafluoroborate								
Bounsiar et al.^5^	283.15–338.15	0.04						
Dreiseitlova et al.^12^	298.15	0.05						
Espiau et al.^16^	298.15	0.07		0.10				
Tomida et al.^19^	293.15–333.15	0.06						

## 5 Summary

This paper presents a thermophysical study at several temperatures (278.15–338.15) K and at atmospheric pressure (0.1 MPa) for three pyridinium-based ILs, (1-butylpyridinium bis(trifluoromethylsulfonyl)imide, 1-hexylpyridinium bis(trifluoromethylsulfonyl)imide, and 1-hexylpyridinium tetrafluoroborate). The following properties were measured: density, speed of sound, refractive index, surface tension, isobaric molar heat capacity, kinematic viscosity, and electrical conductivity. Moreover, some derived properties (isentropic compressibility, molar refraction, and dynamic viscosity) were calculated. These results together with those for 1-butylpyridinium tetrafluoroborate (published previously) have been discussed with the aim of analyzing the effect of both the alkyl chain length of the cation and the anion substitution. The presence of both the tetrafluoroborate anion and a shorter length of the alkyl chain attached to the cation favor both better packing and a higher cohesive energy. On the other hand, the dynamic viscosity of these ILs is more influenced by the nature of the anion. Finally, with respect to the electrical conductivity, the length of the alkyl chain plays the most important role.

## Supporting Information Available

Plots for some properties (density, speed of sound, isentropic compressibility, refractive index, and surface tension) as a function of temperature; figures showing the relative deviations, RD, between our experimental data and those of the literature (PDF)
