# Supplementary material for: Thermophysical Study of Pyridinium-Based Ionic Liquids Sharing Ions
Source: J Chem Eng Data. 2022 Feb 10;67(3):636–43. doi: 10.1021/acs.jced.1c00925 (PMC9997080; doi:10.1021/acs.jced.1c00925)
Supplement: Supplementary file 1 — je1c00925_si_001.pdf [file je1c00925_si_001.pdf]

SUPPORTING INFORMATION

Thermophysical Study of Pyridinium-based Ionic Liquids Sharing Ions

Christian Reinado, Adrián Pelegrina, Miguel Sánchez-Rubio, Héctor Artigas, Carlos Lafuente*

Departamento de Química Física, Facultad de Ciencias, Universidad de Zaragoza, 50009, Zaragoza, Spain.

*Corresponding author. Tel: +34 976762295, E-mail address: celadi@unizar.es

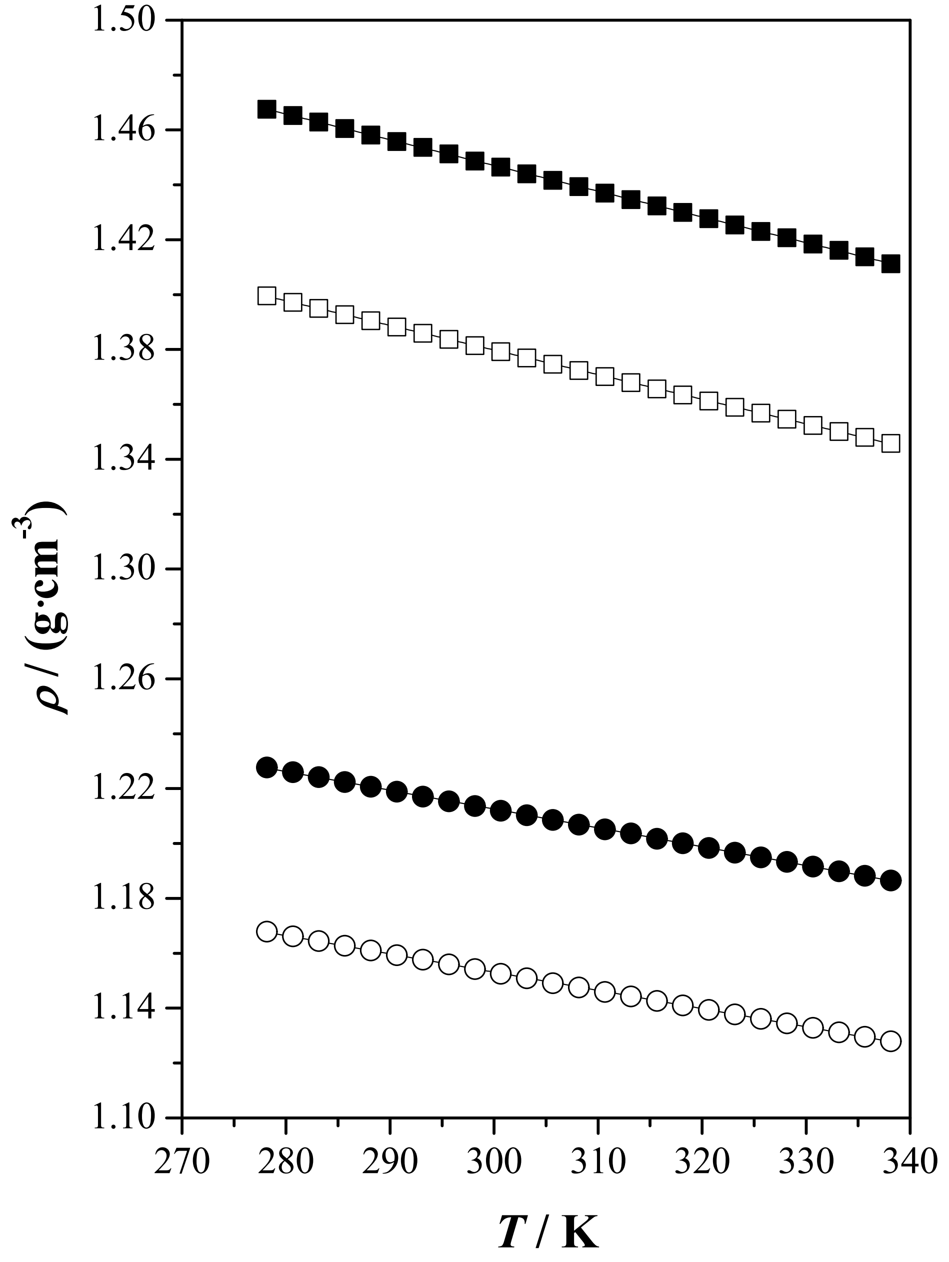

Figure S1. Density, ρ, as a function of temperature, T, at p= 0.1 MPa for the studied ionic liquids and [bpy][BF_4]: (■), [bpy][Tf_2N]; (●), [bpy][BF_4] (Reference 4); (□), [hpy][Tf_2N]; (○), [hpy][BF_4]; (—–) correlated values.

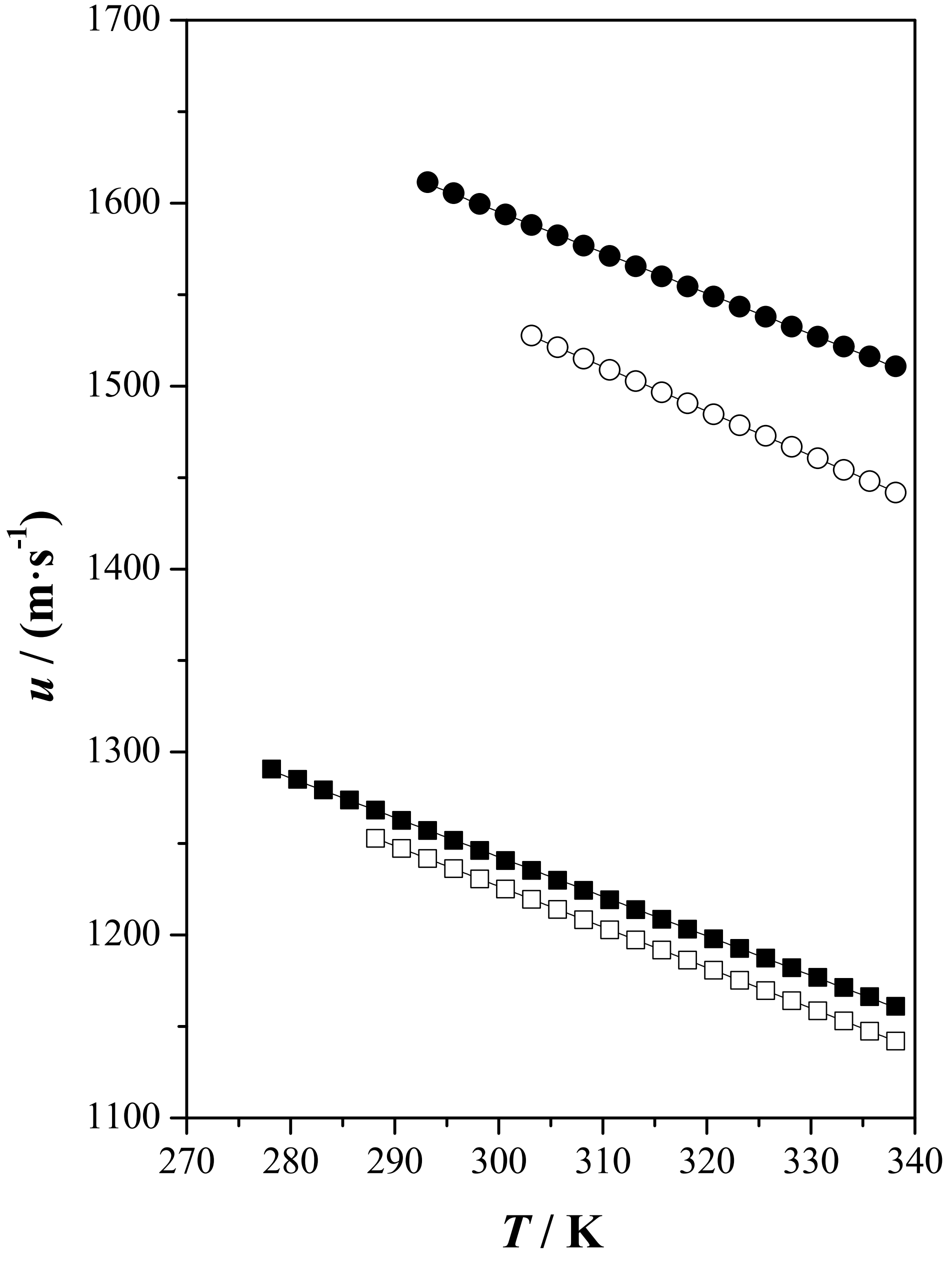

Figure S2. Speed of sound, *u*, as a function of temperature, *T*, at *p*= 0.1 MPa for the studied ionic liquids and [bpy][BF_4]: (■), [bpy][Tf_2N]; (●), [bpy][BF_4] (Reference 4); (□), [hpy][Tf_2N]; (○), [hpy][BF_4] ; (——) correlated values.

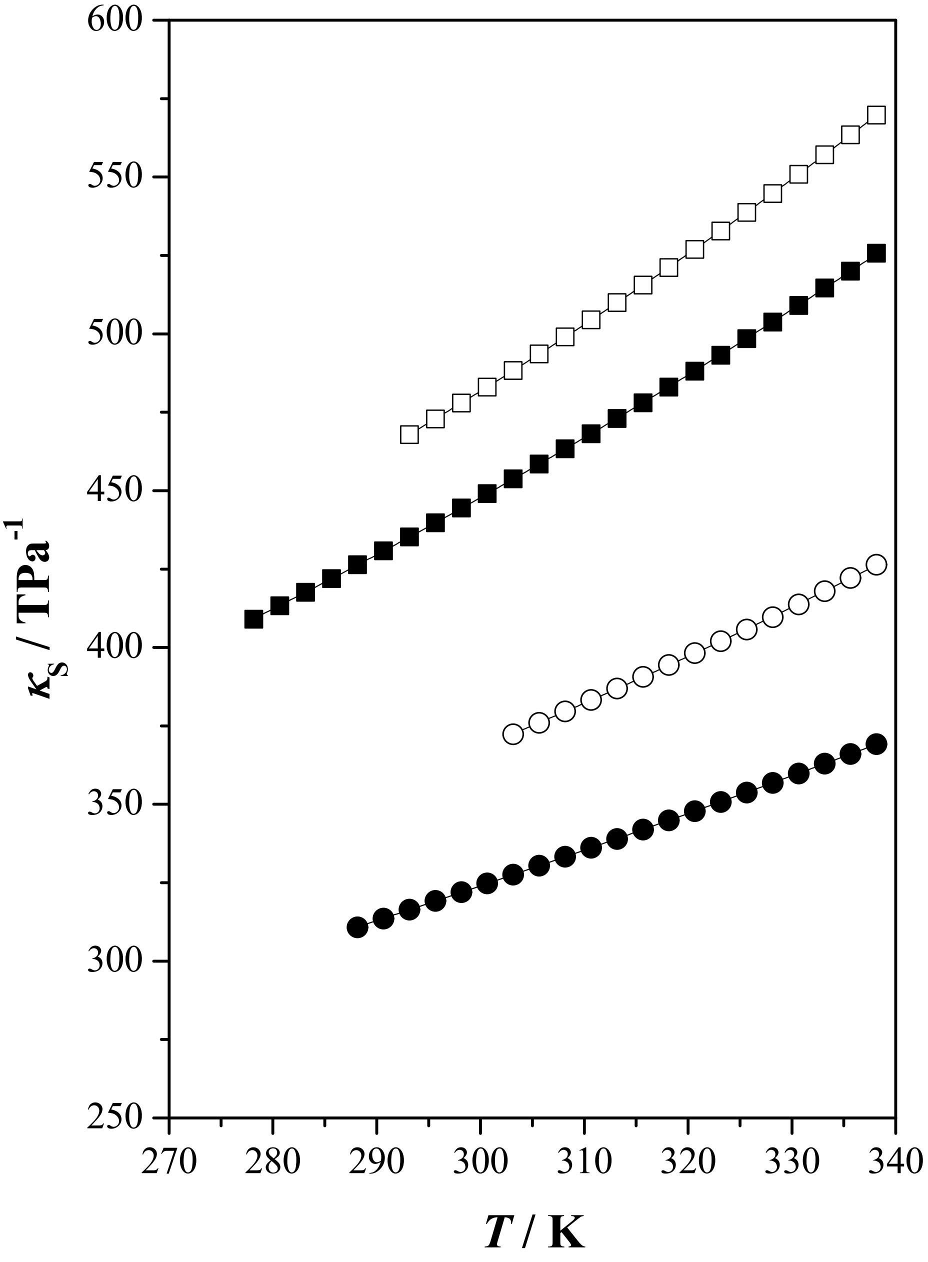

Figure S3. Isentropic compressibility, κ_S, as a function of temperature, T, at p= 0.1 MPa for the studied ionic liquids and [bpy][BF_4]: (■), [bpy][Tf_2N]; (●), [bpy][BF_4] (Reference 4); (□), [hpy][Tf_2N]; (○), [hpy][BF_4] ; (—) correlated values.

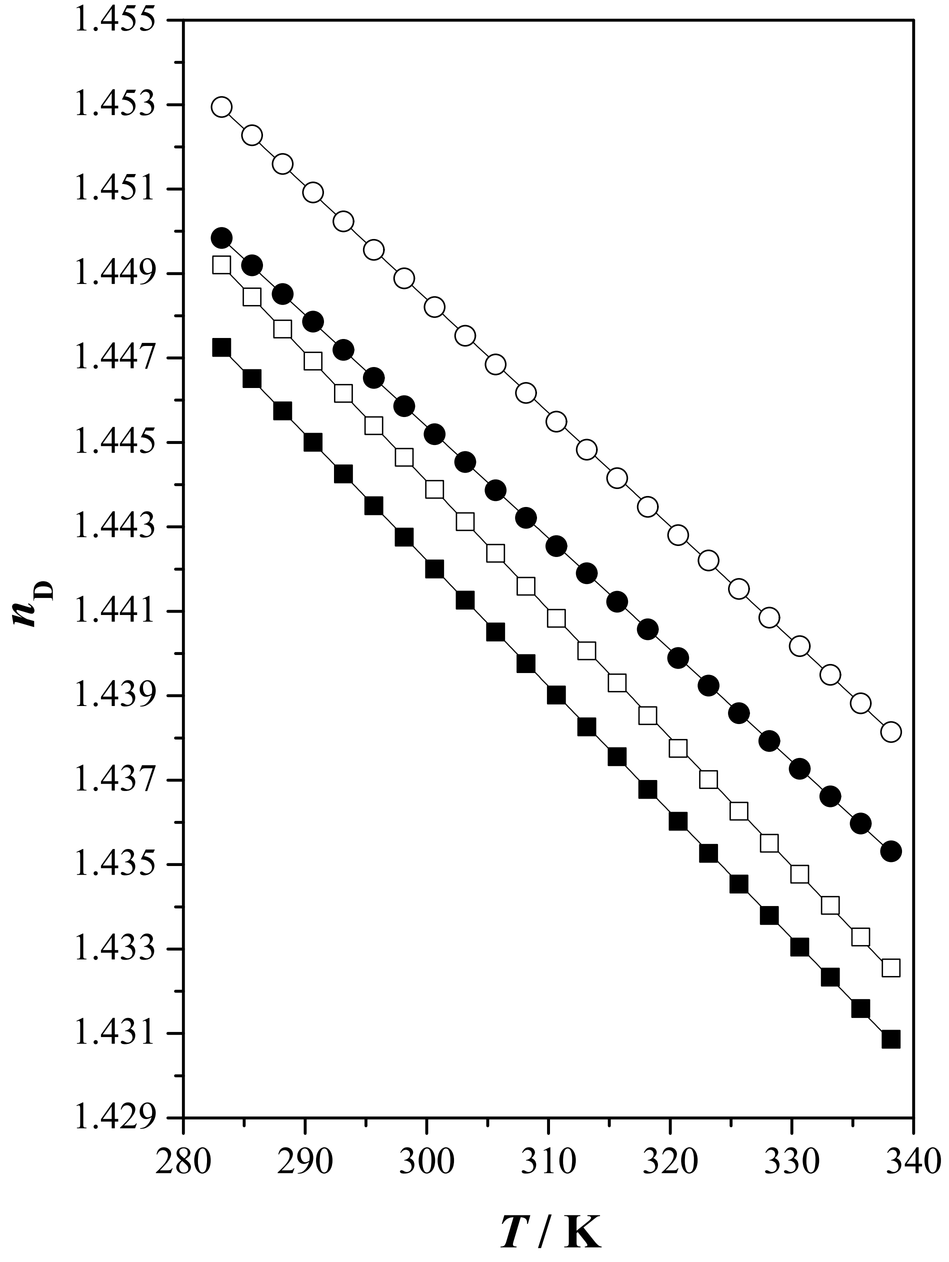

Figure S4. Refractive index, n_D, as a function of temperature, T, at p= 0.1 MPa for the studied ionic liquids and [bpy][BF4]: (■), [bpy][Tf2N]; (●), [bpy][BF4] (Reference 4); (□), [hpy][Tf2N]; (○), [hpy][BF4] ; (—) correlated values.

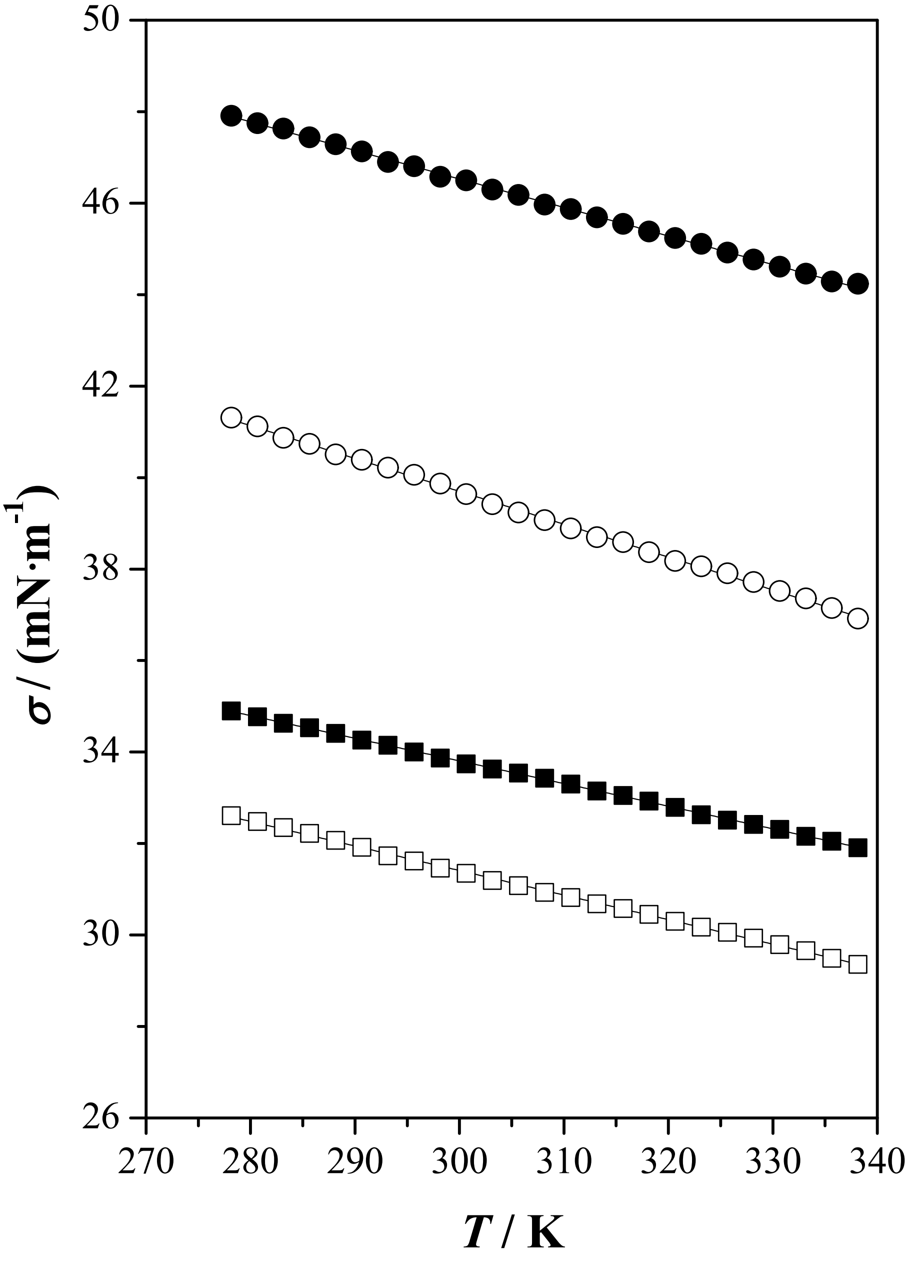

Figure S5. Surface tension, *σ*, as a function of temperature, *T*, at *p*= 0.1 MPa for the studied ionic liquids and [bpy][BF_4]: (■), [bpy][Tf_2N]; (●), [bpy][BF_4] (Reference 4); (□), [hpy][Tf_2N]; (○), [hpy][BF_4] ; (—–) correlated values.

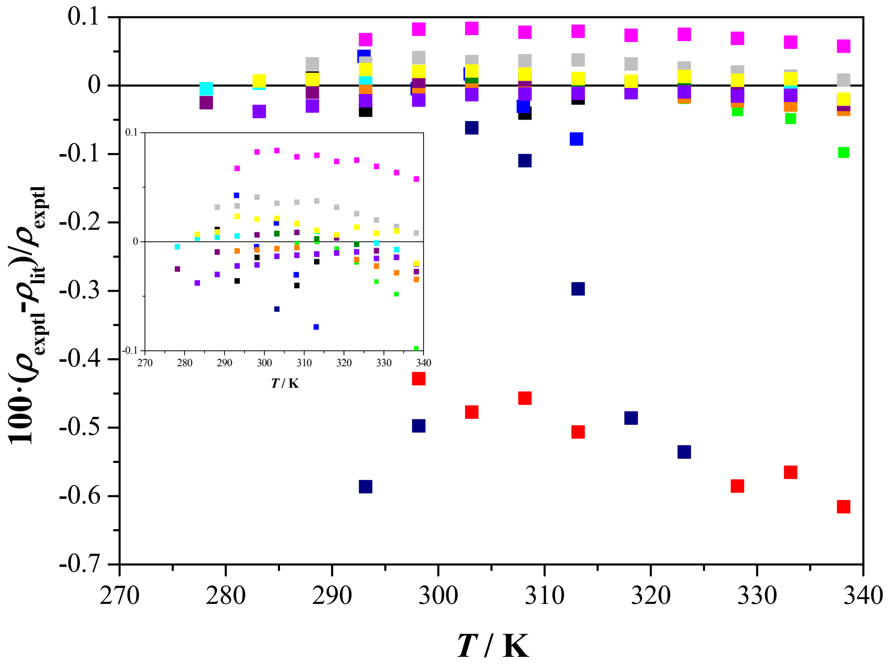

Figure S6. Relative deviations between experimental and literature densities, ρ, as a function of temperature, T, at p = 0.1 MPa for [bpy][Tf_2N]: (■) (Reference 2); (■) (Reference 3); (■) (Reference 5); (■) (Reference 7); (■) (Reference 10); (■) (Reference 11); (■) (Reference 17); (■) (Reference 18); (■) (Reference 21); (■) (Reference 22); (■) (Reference 23); (■) (Reference 24); (■) (Reference 25).

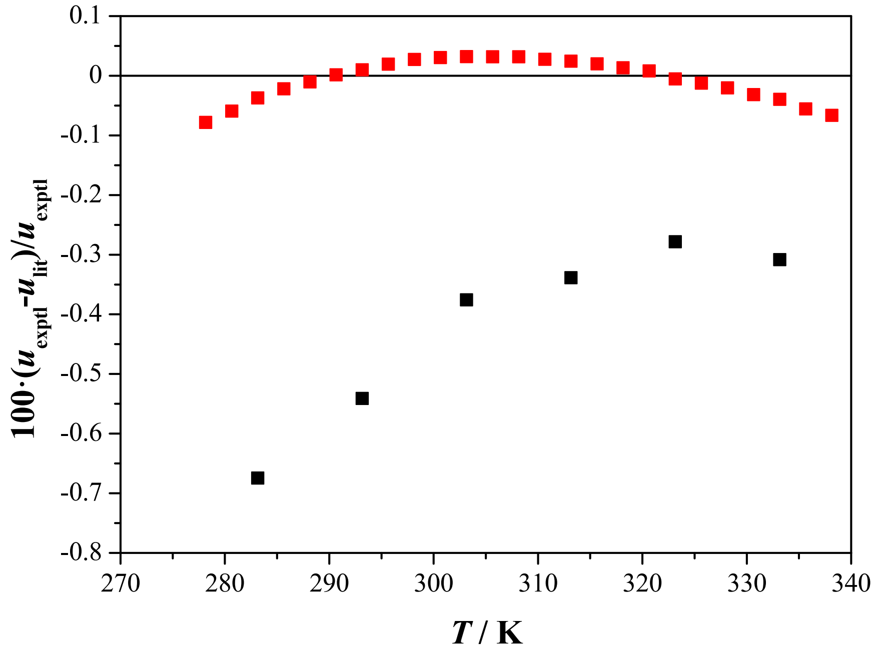

Figure S7. Relative deviations between experimental and literature speeds of sound, u, as a function of temperature, T, at p = 0.1 MPa for [bpy][Tf_2N]: (■) (Reference 24); (■) (Reference 25).

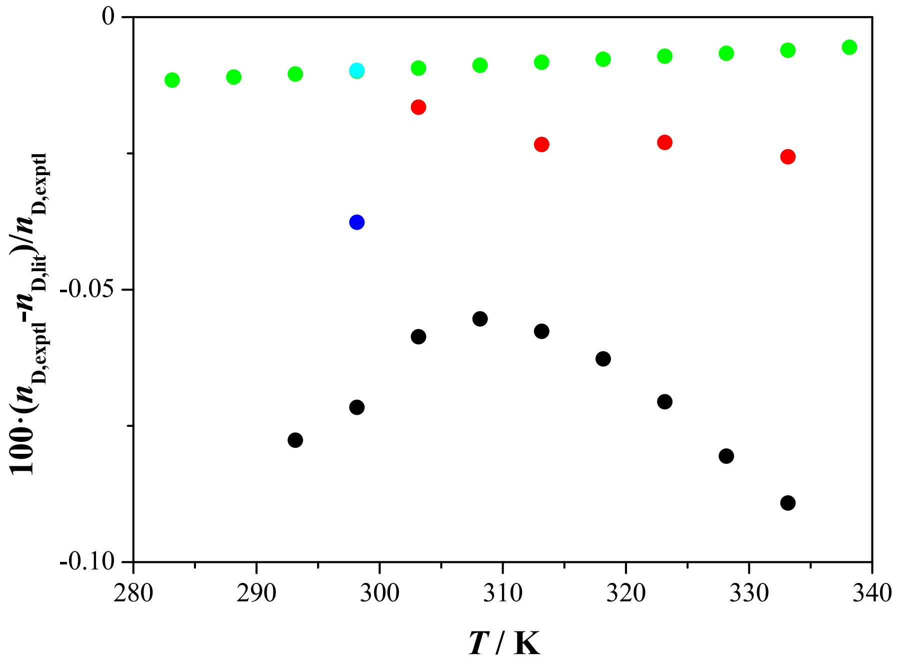

Figure S8. Relative deviations between experimental and literature refractive indices, n_D, as a function of temperature, T, at p = 0.1 MPa for [bpy][Tf_2N]: (■) (Reference 11); (■) (Reference 18); (■) (Reference 23); (■) (Reference 24); (■) (Reference 25).

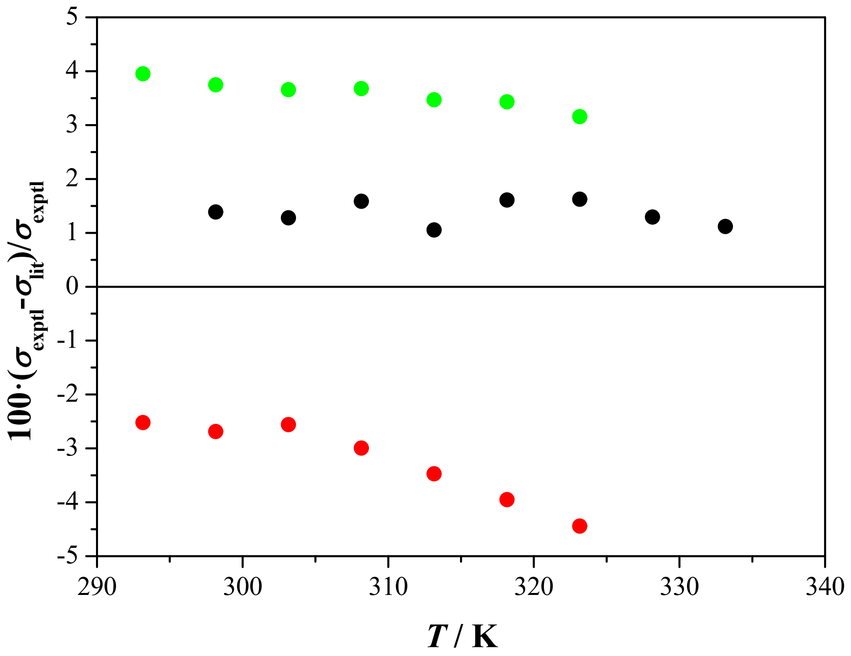

Figure S9. Relative deviations between experimental and literature surface tensions, σ, as a function of temperature, T, at p = 0.1 MPa for [bpy][Tf_2N]: (■) (Reference 3); (■) (Reference 17); (■) (Reference 25).

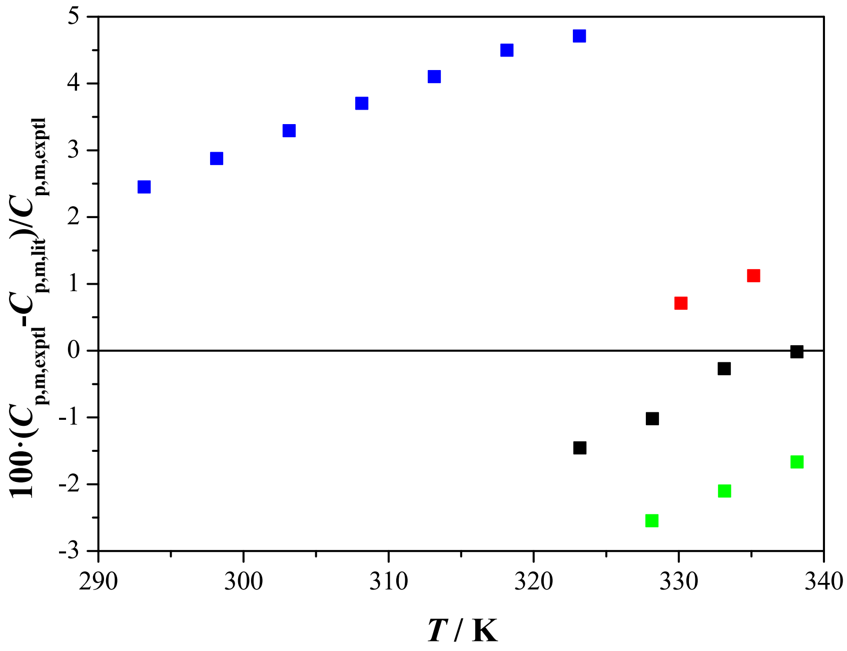

Figure S10. Relative deviations between experimental and literature isobaric molar heat capacities, $C_{p,m}$, as a function of temperature, T, at p = 0.1 MPa for [bpy][Tf_2N]: (■) (Reference 9, large sample scanning calorimetry); (■) (Reference 9, small sample scanning calorimetry); (■) (Reference 9, modulated-temperature scanning calorimetry); (■) (Reference 25).

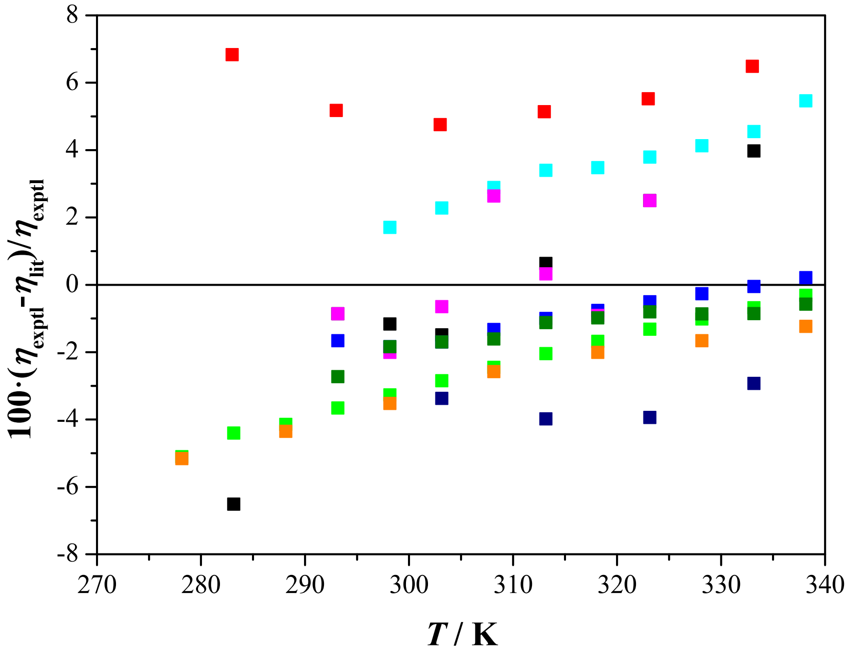

Figure S11. Relative deviations between experimental and literature dynamic viscosities, η, as a function of temperature, T, at p = 0.1 MPa for [bpy][Tf_2N(■) (Reference 2); (■) (Reference 7); (■) (Reference 10); (■) (Reference 11); (■) (Reference 13); (■) (Reference 17); (■) (Reference 18); (■) (Reference 21); (■) (Reference 23).

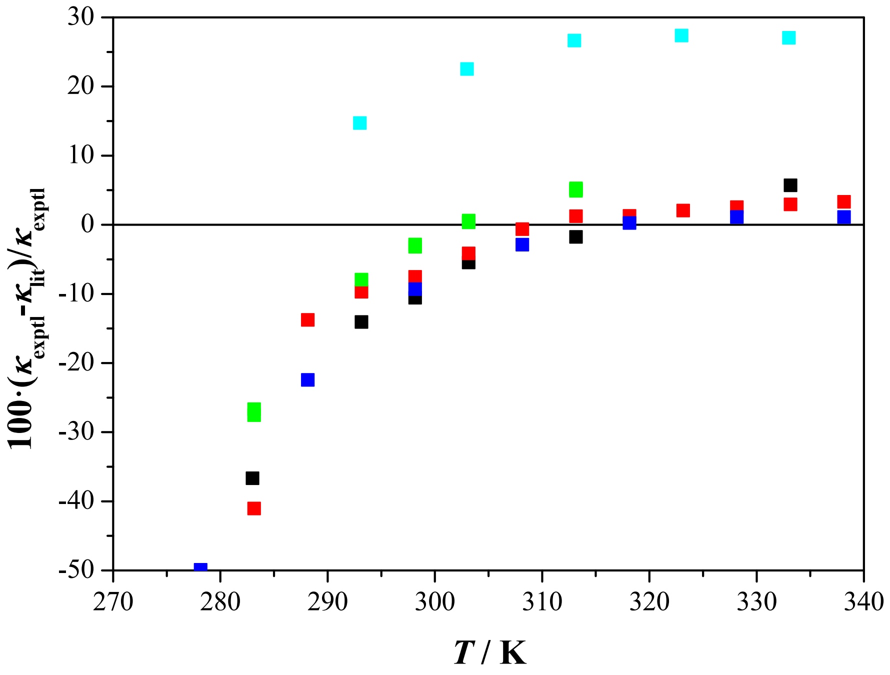

Figure S12. Relative deviations between experimental and literature electrical conductivities, κ, as a function of temperature, T, at p = 0.1 MPa for [bpy][Tf_2N]: (■) (Reference 3); (■) (Reference 17); (■) (Reference 25); (■) (Reference 11); (■) (Reference 18).

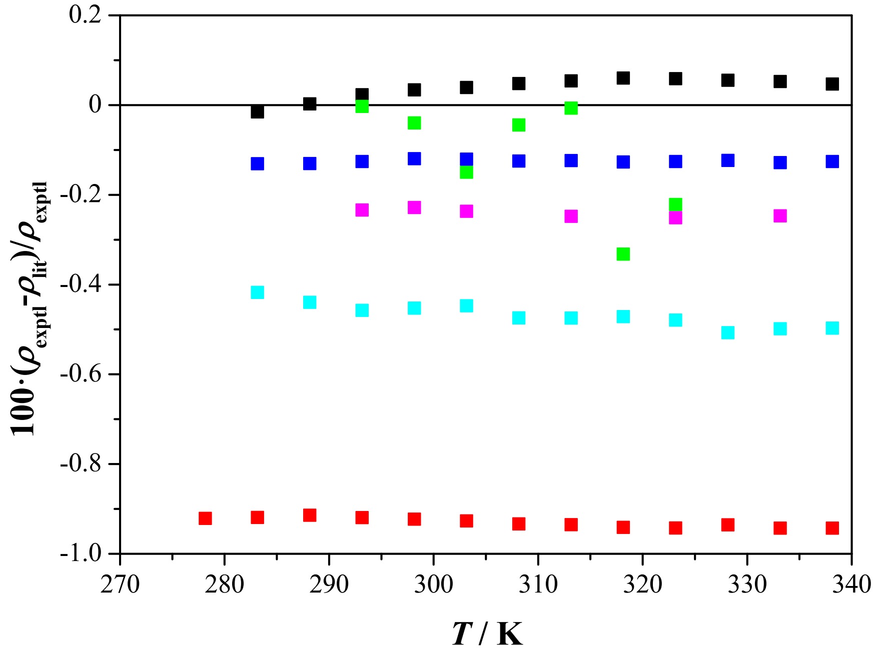

Figure S13. Relative deviations between experimental and literature densities, ρ, as a function of temperature, T, at p = 0.1 MPa for [hpy][Tf_2N]: (■) (Reference 5); (■) (Reference 10); (■) (Reference 17); (■) (Reference 25); (■) (Reference 14) (■) (Reference 20).

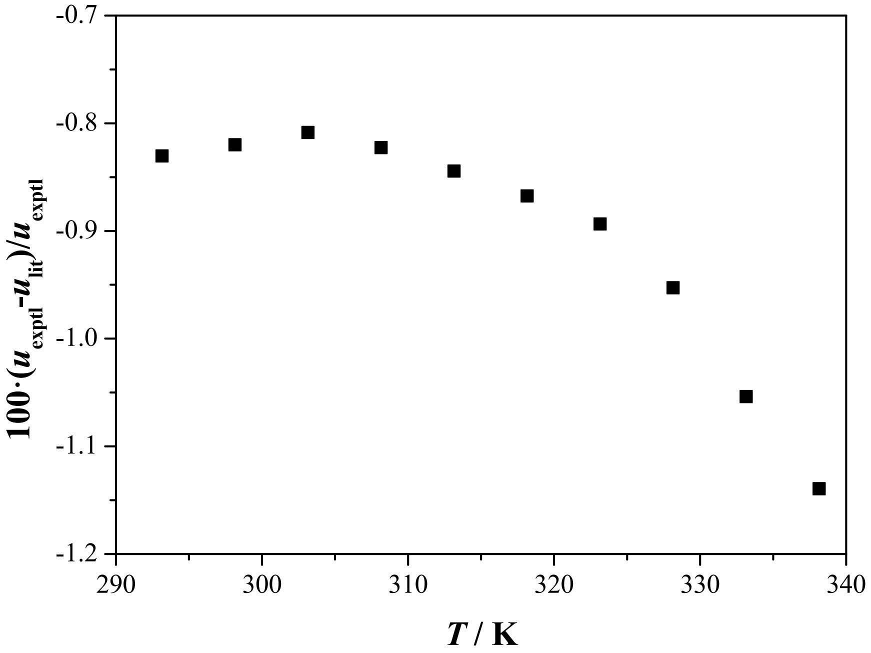

Figure S14. Relative deviations between experimental and literature speeds of sound, u, as a function of temperature, T, at p = 0.1 MPa for [hpy][Tf_2N]: (■) (Reference 25).

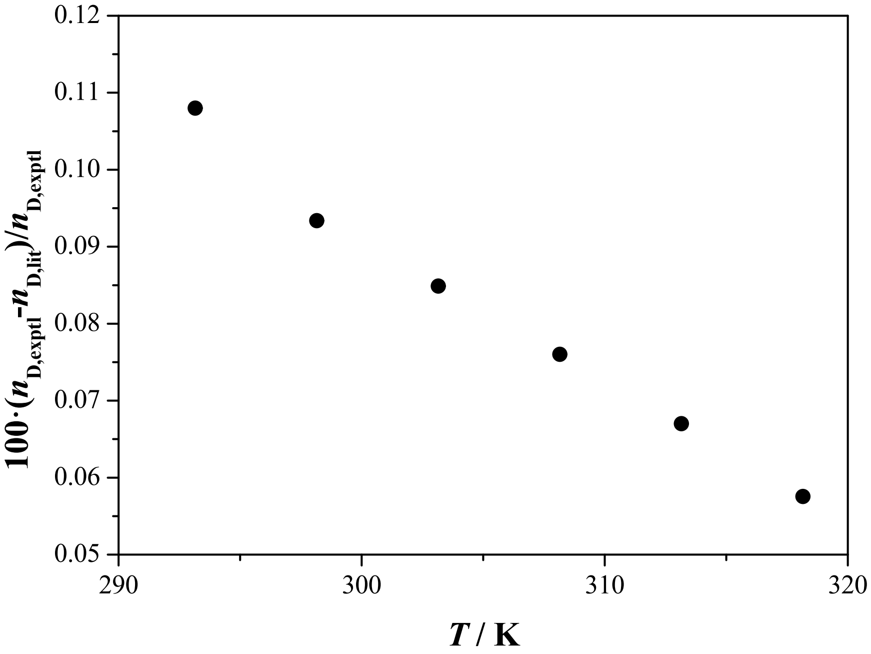

Figure S15. Relative deviations between experimental and literature refractive indices, n_D, as a function of temperature, T, at p = 0.1 MPa for [hpy][Tf_2N]: (■) (Reference 25).

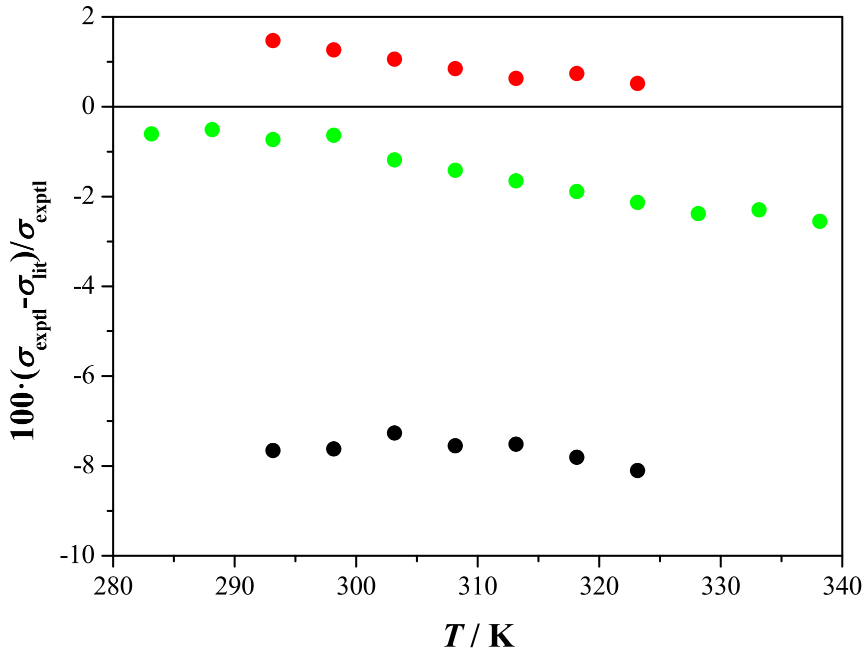

Figure S16. Relative deviations between experimental and literature surface tensions, σ, as a function of temperature, T, at p = 0.1 MPa for [hpy][Tf_2N]: (■) (Reference 17); (■) (Reference 25); (■) (Reference 14).

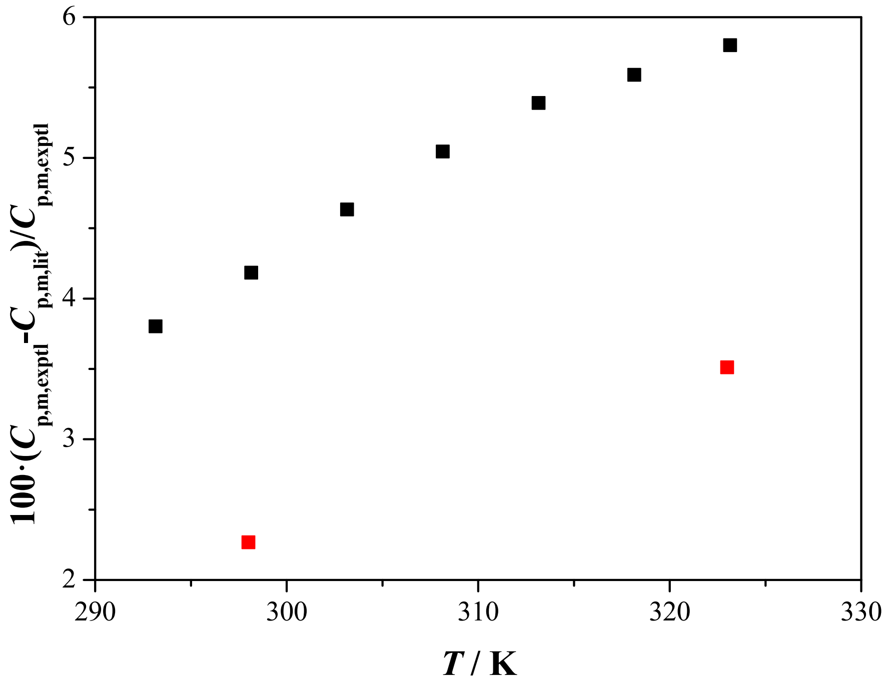

Figure S17. Relative deviations between experimental and literature isobaric molar heat capacities, $C_{p,m}$, as a function of temperature, T, at p = 0.1 MPa for [hpy][Tf_2N]: (■) (Reference 25); (■) (Reference 8).

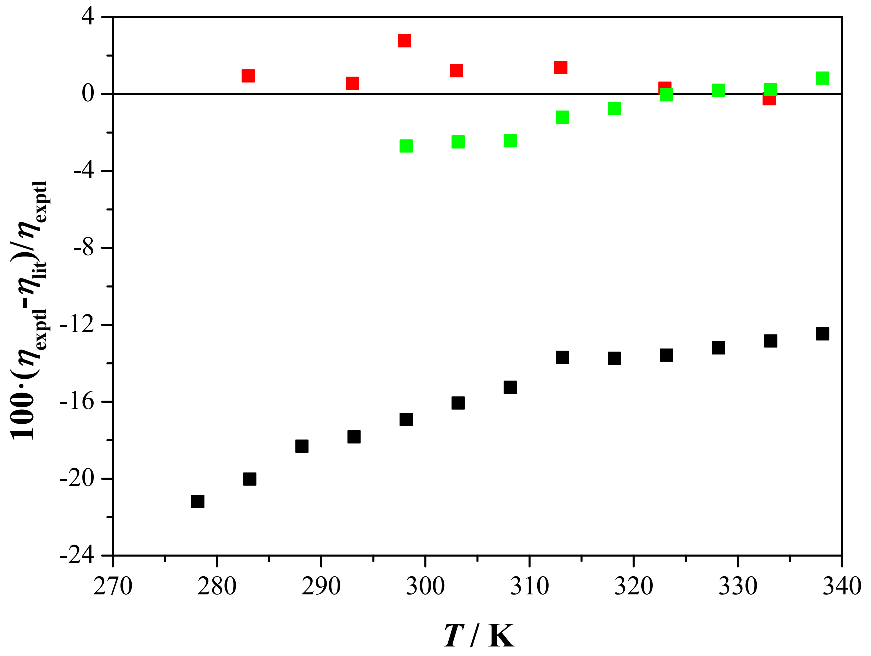

Figure S18. Relative deviations between experimental and literature dynamic viscosities, η, as a function of temperature, T, at p = 0.1 MPa for [hpy][Tf_2N]: (■) (Reference 10); (■) (Reference 8); (■) (Reference 14).

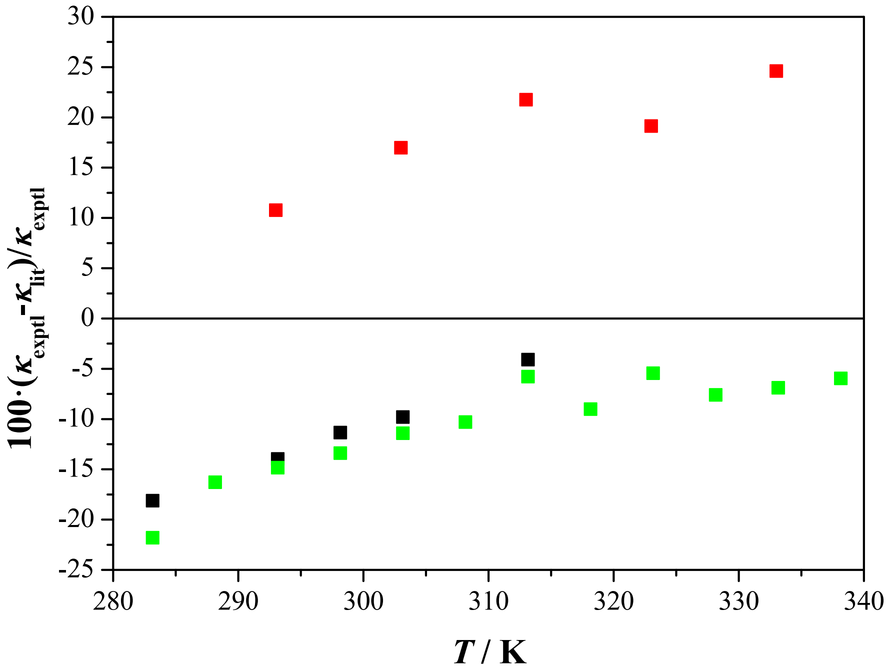

Figure S19. Relative deviations between experimental and literature electrical conductivities, κ, as a function of temperature, T, at p = 0.1 MPa for [hpy][Tf_2N]: (■) (Reference 15); (■) (Reference 25); (■) (Reference 14).

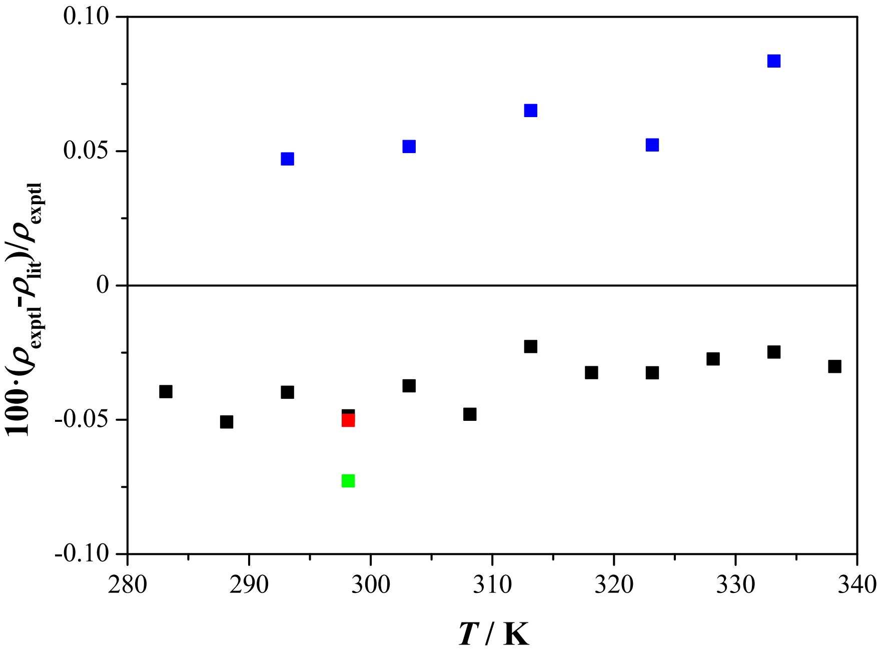

Figure S20. Relative deviations between experimental and literature densities, ρ, as a function of temperature, T, at p = 0.1 MPa for [hpy][BF_4]: (■) (Reference 5); (■) (Reference 12); (■) (Reference 16); (■) (Reference 19).
